# Social Determinants of Health, the Family, and Children’s Personal Hygiene: A Comparative Study

**DOI:** 10.3390/ijerph16234713

**Published:** 2019-11-26

**Authors:** Antonio Jesús Ramos-Morcillo, Francisco José Moreno-Martínez, Ana María Hernández Susarte, César Hueso-Montoro, María Ruzafa-Martínez

**Affiliations:** 1 Department of Nursing, Faculty of Nursing, University of Murcia, 30100 Espinardo, Spain; ajramos@um.es; 2 Murcian Institute of Social Action, 30120 El Palmar, Murcia, Spain; 3 Murcian Health Service, 30007 Murcia, Spain; anadocfisio@hotmail.com; 4 Faculty of Health Sciences, University of Granada, 18016 Granada, Spain; cesarhueso@ugr.es (C.H.-M.); maruzafa@um.es (M.R.-M.)

**Keywords:** child behaviour, child nursing, child protection, family care, inequalities in health, school health services

## Abstract

Habits of personal hygiene are mostly acquired during childhood, and are, therefore, influenced by one’s family. Poor hygiene habits are a risk factor for preventable disease and social rejection. Social Determinants of Health (SDH) consist of contextual factors, structural mechanisms, and the individual’s socioeconomic position, which, via intermediary determinants, result in inequities of health and well–being. Dysfunctional family situations may, therefore, be generated by an unequal distribution of factors determining SDH. Little attention has been paid to the influence of the family on personal hygiene and the perception of social rejection in children. We designed a study to examine differences in personal hygiene and in the perception of social rejection between children in reception centers and children living in a family setting. A validated questionnaire on children’s personal hygiene habits was completed by 51 children in reception centers and 454 children in normal families. Hygiene habits were more deficient among the children in reception centers than among the other children in all dimensions studied. Deficient hygiene habits were observed in the offspring of families affected by the main features of social inequality, who were more likely to perceive social rejection for this reason and less likely to consider their family as the greatest influence on their personal hygiene practices.

## 1. Introduction

Inequalities in health and well-being among social groups have been largely attributed to social determinants of health (SDHs), which are considered at least as important as biological mechanisms for disease prevention and treatment [1]. SDHs include contextual factors and structural mechanisms and the particular socioeconomic status of individuals. Intermediate determinants of SDHs include material resources, psychosocial, behavioral, and biological factors, and healthcare systems. Health inequities are caused by an unequal distribution of factors determining SDH [1].

Multi-level ecological models of SDHs include the family within the “social, family, and community networks” domain, considered not only a source of support and sustenance but also considered an educational resource for the acquisition of healthy habits. Negative aspects are also recognized, with family conflict being a possible risk factor [2]. The family is the first and most important influence on the health and development of children and on the shaping of their routines, habits, attitudes, and social behaviors, including personal hygiene habits [3,4,5].

Improvements in sanitary conditions and the acquisition of certain personal hygiene practices during childhood have played a decisive role in reducing infant mortality and increasing life expectancy [6]. However, diseases related to poor hygiene (e.g., diarrhea or respiratory infections) still kill millions of infants in countries with the greatest social inequalities [7,8,9]. Inadequate hygiene practices have also been implicated in infant morbidity in developed countries, including infectious and parasitic diseases, pneumonia [8], otitis, mycosis, diarrhea, dental caries, gingivitis, and pediculosis [10,11]. Poor hygiene can also be a cause of social rejection, especially for children from poorer families [12].

An inadequate family income is considered a primary cause of poor health in children [13,14], but the role of the family as the social determinant has not been sufficiently considered, even though SDH–related factors are known to affect the capacity of families to care for their children [15]. However, researchers have often analyzed the family in a fragmented manner rather than as a unit. For instance, it has been investigated whether the wealth of families and relationships with parents predict healthy behaviors in young people or whether the parental educational level is associated with personal hygiene habits [16].

In recent years, the risk of family poverty has been increased by economic recession, family breakups, and migration, among other factors [17]. Economic inequalities and the lack of effective social policies have affected the most vulnerable, possibly generating unstructured and dysfunctional families [18]. In extreme cases, such as abuse or abandonment, the state can move children into reception centers for their protection and safety [19]. Children in reception centers (CRCs) have been described as invisible [20], and there has been little research on their health–related lifestyles.

Analysis of the influence of the family as SDH involves the identification of health or healthcare disparity between vulnerable and less-vulnerable populations [21]. The objective of this study was to determine whether CRCs and children living in families (CLFs) differ in their personal hygiene habits and learning and in their perception of social rejection.

## 2. Materials and Methods

### 2.1. Design

This observational, cross-sectional study compares a group of CRCs with a group of CLFs.

### 2.2. Setting and Participants

The study was carried out in the Murcia Autonomous Community (Spain). This is located in the south–east region of the Iberian Peninsula. It covers 11,314 km^2^ and has a population density of 130.63 inhabitants/km^2^. On 1 January 2018, the population was 1,475,568 inhabitants. The gross monthly average salary of Murcia Autonomous Community workers is 1.761 euros, life expectancy at birth is 82,37 years old, and the unemployment rate was 14.16% [22]. In 2016, there were 502 primary schools and 80% public schools, which means there were 25,939 students [23].

Children aged between 7 and 12 years old were studied from March 2015 through January 2017. CRCs were recruited within the first two days of admission to two centers in the Network of State Care Centers (first stage in the fostering process) of Murcia Autonomous Community, whose main function is to offer immediate temporary shelter and protection to minors who have been abandoned or are at high social risk. CLFs were recruited from three primary public schools selected by convenience from a rural (<30,000 inhabitants), suburban (30,000–50,000 inhabitants), and urban (>50,000 inhabitants) setting in the same region.

### 2.3. Sample Selection

The eligible population was all children in the selected schools and reception centers who met the following inclusion criteria: age between 7 and 12 years, voluntary participation, and written consent to participate from parents or legal guardians. Exclusion criteria were inability to speak Spanish or the presence of physical/psychological disabilities that hindered participation. CRCs who had previously been admitted to care were also excluded to avoid the influence of hygiene habits acquired during earlier admission(s).

### 2.4. Variables and Measurement Instruments

A questionnaire was administered to parents/guardians of the CLFs to gather data on their educational level, current occupation, type of employment, and household monthly income. CLFs were divided into three groups according to income: ≤1000 €, 1001–2000 €, and >2000 €. Information was also collected on the sex, age, and nationality of the children, the nationality of the parents, the number of siblings, and the days of school attendance per week.

The outcome variables considered were: body, hair, hands, and oral hygiene, agents that influence personal hygiene learning, perceived social rejection, and motivations for personal hygiene activities. These data were gathered using the HICORIN^®^ questionnaire, validated for Spanish populations, which has demonstrated adequate reliability and validity [24]. It includes 63 items divided among seven personal hygiene dimensions and hygiene-related social aspects. For this study, we selected items related to the frequency, manner, and timing of personal hygiene activities and the materials used, considering the following dimensions: body skin (8 items), hair (2 items), hands (5 items), oral (14 items), agents affecting personal hygiene learning (8 items), social rejection (2 items), and motivation for personal hygiene activities (5 items).

### 2.5. Data Collection

The HICORIN^®^ questionnaire was administered by the interviewer for children aged 7–10 years and self–administered for those aged 11–12 years. It was completed by participating CLFs at school one week after consent to participation was obtained from parents/guardians and by participating CRCs within two days of admission to the reception centers. Economic data for the parents/guardians of CRCs were gathered from the computer records of the protection centers.

### 2.6. Ethical Considerations

The study was conducted in accordance with the Declaration of Helsinki, and the protocol was approved by the Research Ethics Committee of University of Murcia (13/12/2013, Code: 220114). Authorization for the children’s participation was obtained from parents/guardians in the case of CLFs and from the General Directorate of Social Policy of Murcia Autonomous Community in the case of CRCs.

### 2.7. Data Analysis

In a descriptive analysis, we calculated absolute and relative frequencies for qualitative variables and means with standard deviation (SD) for quantitative variables. We used the chi-square test to assess differences in the personal hygiene habits between CRCs and CLFs by stratifying CLF families into three income levels. To identify the contribution of different cells to the significance of this chi-square, we used adjusted standardized residuals, considering statistically significant for those greater than +/−2 [25]. We performed multivariate binomial logistic regression analysis to compare the prevalence of hygiene habits between CRCs and CLFs, calculating crude odds ratios (ORs), and ORs adjusted for sex and age with a 95% confidence interval (95% CI). IBM SPSS 21.0 (IBM SPSS, Chicago, IL, USA) was used for the data analysis, considering *p* < 0.05 to be statistically significant.

## 3. Results

Study eligibility criteria were met by 51 children admitted to the two reception centers during the study period, and all completed the questionnaire (100% response rate). Furthermore, 47% are male and are 9.9 (standard deviation 0.27) years old. Eligibility criteria were met by 758 CLFs in the three schools, and 454 of them completed the questionnaire (59.89% response rate). In addition, 50% are male and are 9.1 (SD 0.07) years old.

### 3.1. Sociodemographic Characteristics of Families and Children

A comparison of the characteristics of the families from the four study groups (CRCs and three groups of CLFs by monthly household income) revealed significant differences in all study variables (Table 1).

In families of CRCs, 90.2% (n = 46) of parents had no schooling or only primary schooling, 48% (n = 24) were employed (50% of these in unskilled work), and 88.2% (45) of the families had a monthly household income ≤1000 €.

A significantly higher percentage of CRCs (21%, n = 11) were immigrants in comparison to the three groups of <CLFs, 41.2% (n = 21) had immigrant mothers and 33% (n = 17) had immigrant fathers, 70.6% (n = 36) had ≥3 siblings, and 31.4% (n = 16) reported not going to school every day (Table 2).

### 3.2. Personal Hygiene Habits

Table 3 exhibits the results for personal hygiene habits. Statistically significant differences were found between CRCs and CLFs in all items. The frequency of body washing “≥3 days a week” was 12-fold to five-fold lower in CRCs than in CLFs. A wet towel/sponge was used for body washing by 23.5% (n = 12) of CRCs versus almost 100% of CLFs who reported taking a shower/bath. Body washing was performed at night (before bedtime) by 39.2% (n = 20) of CRCs versus <50% of CLFs. With regard to materials, gels were not used or known by 21.6% (n = 11) of CRCs versus 3.6–7.1% of CLFs. The use of bar soap was uncommon but was more frequent by CRCs than by CLFs (17). A washbowl was more often used in body washing by CRCs than by CLFs, whose use of this complement was less frequent with higher household income. A hair washing frequency of “≥3 times/week” was 8.6–9.5–fold lower in CRCs than in the CLF groups. The frequency of shampoo use did not significantly differ between CRCs and CLFs (*p* = 0.364).

As shown in Table 4, “hand washing ≥3 times a day” was reported by a lower percentage of CRCs than of CLFs, regardless of their household income, but the difference was not statistically significant. The use of soap in hand washing was significantly less frequent among CRCs (39.2%, 20) than among CLFs in the income group between 1001 and 2000 € (89.2%, 91). Statistically significant differences were also obtained in hand washing frequencies, which were always lower for CRCs. Hand washing after defecating was the most frequent practice in all study groups, even though it was reported by a higher percentage of CLFs than CRCs.

Statistically significant results were observed in all oral hygiene items (Table 5). The tooth brushing frequency “≥2 times a day” was reported by a lower percentage of CRCs than of CLFs, and the percentage of CLFs increased with higher household incomes. The highest frequency of tooth brushing by all of the children was at night (before bedtime). However, it was significantly less frequent in CRCs. With respect to the materials used for oral hygiene, toothbrush and toothpaste were the most frequently used (>80% in all groups). In addition, 17.6% (9) of CRCs shared their toothbrush with other family members. There were also significant differences in the type of toothbrush used, with 6% of CRCs reporting the use of an electrical toothbrush versus 40% of CLFs. Conversely, utilization of a toothpick was reported by a higher percentage of CRCs than of CLFs (37.3%, 19). The frequency of dentist visits during the previous year was significantly lower in CRCs and was greater with higher household income in CLFs.

### 3.3. Personal Hygiene Practice Learning and Social Rejection

The family (father, mother, or other family member) was most frequently described by all groups of children as having the greatest influence on their personal hygiene learning. However, this affirmation was made by a significantly lower percentage of CRCs (72.5%, 37) than of CLFs. In second place as learning agents, CLFs selected healthcare professionals, while CRCs selected teachers, radio, television, Internet, and self-learning (Table 6).

A significantly higher proportion of CRCs (41.1%) had experienced social rejection for being dirty in comparison to the CLFs (9.5% of those with family incomes <1000 € and around 4% of those with higher family incomes). Very similar differences were observed in the experience of rejection for smelling poorly. Yet, the percentages were slightly higher in all groups, reaching almost 50% in CRCs and between 8.9% and 10.7% in CLFs. The only statistically significant difference in motivations for personal hygiene activities was the option “to not be rejected by friends,” which was selected by 90% of CRCs versus 58% of CLFs from families with incomes <1000 € and 36% of those from families with higher incomes.

## 4. Discussion

There has been little research in developed countries on SDHs related to family and personal hygiene in childhood, and this study, therefore, contributes important empirical data. The main finding was a clear relationship between the personal hygiene of the children and their family settings.

In this study, CLFs were compared with CRCs whose situation of vulnerability was sufficiently extreme to warrant removal from their families [26]. The parents/guardians of the CRCs exhibit the main axes of inequality [1], which is characterized by a low schooling level, low qualifications, unemployment or only casual unskilled employment, and an income <1000 €, with 70% having ≥3 children. Among the CRCs, 78.4% were born in Spain. However, around two out of five of their parents were immigrants, and almost one out of three children did not go to school every day. Stratified comparison by incomes were useful to determine if CRCs and CFL in families with the lowest level of income could share a relatively similar condition of well–being. Both groups (CRCs and CFL Incomes <1000 €) have elevated rates of low incomes, unemployment, and immigration, but percentages of no or primary studies, unqualified workers, number of siblings, and less days of school attendance increase at CRCs.

In comparison to the CLFs, the CRCs had poorer hygiene habits in all dimensions studied, were less likely to consider their families as the most influential agent in learning hygiene habits, and were more likely to experience social rejection due to their hygiene and to be motivated by this rejection to carry out personal hygiene activities. These findings question whether vulnerable families are adequately fulfilling their functions of protection, healthcare, and socialization [21,27].

The lower frequency of key personal hygiene practices (body, hair, and hand washing and tooth brushing) in the children from vulnerable families is consistent with reports that implicate the family structure and low parental educational and socioeconomic level in poor health, hygiene [28], and oral hygiene [29] behaviors in children. A strong relationship has been reported between a low level of healthy habits and worsening children’s health as perceived by their mothers (OR = 0.48 95% CI = 0.42–0.56) [28]. Hence, the detection of signs of poor personal hygiene practices in children may help professionals anticipate situations of ill health.

As previously observed by Wagstaff [30], an association was found between lower family income and worse hygiene practices, especially dental hygiene habits, with less frequent daily tooth brushing, tooth brushing before bedtime, and visits to the dentist. Despite the offer of free oral healthcare, visits to the dentist during the previous year were between 10-fold and 20.7-fold less frequent for CRCs than for CLFs. Researchers have concluded that families with lesser resources have a worse relationship with healthcare systems [18,31], which they engage with to a lesser extent, in part due to the lack of recognition of health problems [32].

The relationship between the physical environment in which children develop and healthy behaviors has also been demonstrated [3,4,5,33]. In the present study, children of vulnerable families were less likely to use a toothbrush (especially an electrical device, that a significant benefit has demonstrated [34]), hand-washing soap, shower gel, or toothpaste, while one in four of them used a washbowl and wet towel or sponge for body washing, which reflects the lack of opportunity to take a bath or shower.

Surprisingly, hand washing was not an established routine in the children, regardless of their family or institutional setting, possibly because it was carried out without the supervision of parents, despite being recommended as a family activity [35]. There was a higher frequency of hand washing after defecation in both CRCs and CLFs, even though it continued to be low in the former. This is similar to the findings of a study in 11 developing countries [36]. This practice requires special attention, given that education in hand washing has been reported to reduce cases of diarrhea by 31% and cases of respiratory diseases by 21% [9,37].

Besides the biological implications of our findings, they are also relevant from a social standpoint, which confirms the value of the family as a key factor in the acquisition of healthy habits [38]. Almost all CLFs and three-quarters of CRCs considered their families to be the main agents for learning hygiene practices, with a role being attributed by CLFs to healthcare professionals and by CRCs to teachers, self–learning, and the mass media, which may reflect the worse relationship of poorer families with healthcare systems [18,31]. These findings suggest the need for educators and healthcare professionals to work together in the design and implementation of strategies to improve the hygiene habits of children from vulnerable families.

Weaknesses in the socialization function of the family were also revealed by the CRCs, who were more likely to be rejected for being dirty or smelling poorly and to be motivated in their personal hygiene activities by the need to avoid this rejection. Hygiene behaviors play an important role in the impression that we make on others and display respect for social norms [12], which facilitates the integration and socialization of children among their peers. There is a relationship among family vulnerability, diseases associated with poor hygiene (e.g., caries and pediculosis), school problems, and marginalization [16,39,40]. The long–term social effects of this situation are unclear. However, it appears likely to perpetuate the inequality and vulnerability of these children, with negative consequences for their health in adulthood [41].

According to the Model of Health Promotion of the Family [38], the values, targets, and needs of families mediate between their health practices and socioeconomic status. Hence, despite the limited influence of nursing professionals on the SDHs affecting families, they may be able to intervene to improve health and hygiene practices. Family interventions have achieved improvements in the acquisition of healthy lifestyles related to physical activity and sport [42] and might, therefore, have a similar impact on personal hygiene habits.

### Study Limitations

The sample size of children from vulnerable families was reduced due to difficulties in gaining access to this relatively small population, even though it proved possible to obtain significant differences with the children living at home. There may have been a “volunteer” bias, given the response rate of only 60% for the CLFs. The study design means that causality relationships could not be established. Community setting (rural/suburban/urban) was not collected individually, so its influence could not be controlled at the multivariable analysis. Lastly, the lack of published data on this issue, especially in developed countries, limited the discussion of our findings.

## 5. Conclusions

Our study provides new evidence on the relationship among SDHs, family, and the personal hygiene practices of children. Our findings raise questions about the adequate fulfillment by vulnerable families of their protection, healthcare, and socialization functions. The results confirm that the family, understood as a complex system that acts on the health behaviors of the individuals that form it, affects the personal hygiene practices of children. Thus, deficient hygiene habits were observed in the offspring of families affected by the main features of social inequality, who were more likely to perceive social rejection for this reason and less likely to consider their family as the greatest influence on their personal hygiene practices.

These findings indicate that the influence of contextual factors and structural mechanisms, which result in the individual’s socioeconomic position and are, therefore, not to be underestimated in a sustainable amelioration of an individual’s health and well-being. Action against social inequality can have a potential impact on biological mechanisms that affect health. Although this inequality cannot be resolved within the family setting, it can be ameliorated by promoting family practices designed to improve personal hygiene habits.

## Figures and Tables

**Table 1:** Comparison of family socioeconomic variables between children in reception centers and those living with their family according to monthly income (N = 455).

Family Socioeconomic Variables	CRC n = 51	CLF Income <1000 € n = 82	CLF Income 1001 €–2000 € n = 97	CLF Income >2000 € n = 225	CLF Total n = 454
Educational level *					
No or primary studies n (%)	46(90.2) ^‡^	37(45.7) ^‡^	18(17.6)	0(0.0) ^‡^	55(13.5)
First stage of secondary schooling or mid–level vocational training n (%)	3(5.9)	8(9.9) ^‡^	9(8.8)	4(1.8) ^‡^	21(5.2)
Second stage of secondary education or higher–level vocational training n (%)	2(3.9) ^‡^	27(33.3) ^‡^	34(33.3) ^‡^	16(7.1) ^‡^	77(18.9)
University studies n (%)	0(0.0) ^‡^	9(11.1) ^‡^	41(40.2) ^‡^	204(91.1) ^‡^	254(62.4)
Current occupation *					
Student, exclusive dedication n (%)	0(0.0)	4(4.8) ^‡^	0(0.0)	0(0.0) ^‡^	4(1.0)
Self–employed n (%)	2(4.0) ^‡^	7(8.4) ^‡^	14(13.7)	54(24.1) ^‡^	75(18.3)
Employed n (%)	20(40.0) ^‡^	34(41.0) ^‡^	54(52.9)	143(63.8) ^‡^	231(56.5)
Retired n (%)	0(0.0)	0(0.0)	1(1.0)	2(0.9)	3(0.7)
Unemployed n (%)	26(52.0) ^‡^	36(43.4) ^‡^	27(26.5)	21(9.4) ^‡^	84(20.5)
Other n (%)	2(4.0)	2(2.4)	6(5.9)	4(1.8)	12(3.0)
Type of employment *					
Manager/director in public administration or and companies with ≥10 workers n (%)	0(0.0) ^‡^	0(0.0) ^‡^	3(4.3) ^‡^	93(47.2) ^‡^	96(31.4)
Manager in companies with <10 workers n (%)	0(0.0)	0(0.0) ^‡^	9(13.0)	30(15.2) ^‡^	39(12.7)
Administrative workers. Personnel services & safety n (%)	2(9.1)	7(17.9)	23(33.3) ^‡^	44(22.3)	74(24.6)
Self–employed n (%)	1(4.5)	9(23.1)	14(20.3)	24(12.2)	47(15.4)
Supervisors of manual workers n (%)	0(0.0)	0(0.0)	1(1.4)	0(0.0)	1(0.3)
Qualified and semi–qualified manual workers n (%)	8(36.4) ^‡^	14(35.9) ^‡^	15(21.7) ^‡^	5(2.5) ^‡^	34(11.1)
Unqualified workers n (%)	11(50.0) ^‡^	9(23.1) ^‡^	4(5.8)	1(0.5) ^‡^	14(4.5)

CRC: children in reception centers, CLF: children living in a family. * *p* < 0.0001 ^^‡^^ Significant cell.

**Table 2:** Comparison of socio–family variables between children in reception centers and those living with their family according to monthly income (N = 455).

Socio-Family Variables		CRC n = 51	CLF Income <1000 € n = 82	CLF Income 1001 €–2000 € n = 97	CLF Income >2000 € n = 225	CLF Total n = 454
Sex	Male n (%)	24(47.1)	39(46.4)	47(47.5)	122(54.2)	226(50.2)
	Female n (%)	27(52.9)	45(53.6)	52(52.5)	103(45.8)	224(49.8)
Age *	7–8 years n (%)	13(26.0)	30(35.7)	37(36.6)	92(40.9)	173(38.3)
	9–10 years n (%)	15(30.0)	36(42.9)	43(42.6)	83(36.9)	181(40.0)
	11–12 years n (%)	22(44.0) ^‡^	18(21.4)	21(20.8)	50(22.2)	98(21.7)
Place of birth **	Spain n (%)	40(78.4) ^‡^	72(84.7) ^‡^	99(97.1)	221(98.2) ^‡^	392(94.7)
	Another country n (%)	11(21.6) ^‡^	10(11.8) ^‡^	2(2.0)	4(1.8) ^‡^	19(4.2)
	Unknown n (%)	0(0.0)	3(3.5) ^‡^	1(1.0)	0(0.0) ^‡^	5(0.8)
Place of birth of mother **	Spain n (%)	30(58.8) ^‡^	59(70.2) ^‡^	94(92.2) ^‡^	213(94.7) ^‡^	366(89.1)
	Another country n (%)	21(41.2) ^‡^	19(22.6) ^‡^	5(4.9) ^‡^	7(3.1) ^‡^	31(7.5)
	Unknown n (%)	0(0.0)	6(7.1) ^‡^	3(2.9)	5(2.2)	14(3.4)
Place of birth of father **	Spain n (%)	33(64.7) ^‡^	59(71.1) ^‡^	91(89.2)	211(93.8) ^‡^	361(88.0)
	Another country n (%)	17(33.3) ^‡^	19(22.9) ^‡^	7(6.9)	4(1.8) ^‡^	30(7.3)
	Unknown n (%)	1(2.0)	5(6.0)	4(3.9)	10(4.4)	19(4.6)
Number of siblings **	None n (%)	2(3.9)	6(7.1)	6(5.9)	13(5.8)	25(6.1)
	1 or 2 n (%)	13(25.5) ^‡^	65(76.5)	82(80.4)	188(83.6) ^‡^	335(81.3)
	3 or more n (%)	36(70.6) ^‡^	14(16.5)	14(13.7)	24(10.7) ^‡^	52(12.6)
School attendance **	Always n (%)	35(68.6) ^‡^	82(98.8)	101(100) ^‡^	224(99.6) ^‡^	407(99.6)
	3 or 4 days a week n (%)	5(9.8) ^‡^	1(1.2)	0(0.0)	0(0.0) ^‡^	1(0.2)
	1 or 2 days a week n (%)	4(7.8) ^‡^	0(0.0)	0(0.0)	1(0.4)	1(0.2)
	Never n (%)	7(13.7) ^‡^	0(0.0)	0(0.0)	0(0.0) ^‡^	0(0.0)

CRC: Children in reception centers. CLF: children living in a family. * *p* < 0.05. ** *p* = 0.001. ^^‡^^ Significant cell.

**Table 3:** Comparison of body and hair hygiene variables between children in reception centers and those living with their family according to monthly income (N = 455).

Body Washing		CRC n = 51	CLF Income <1000 € n = 82	CLF Income 1001 €–2000 € n = 97	CLF Income >2000 € n = 225	CLF Total n = 454
Weekly Frequency ***	>3 days n (%)	26 (51.0)	76 (89.4)	93 (92.1)	200 (90.5)	369(90.7)
	<2 days n (%)	25 (49.0)	9 (10.6)	8 (7.9)	21 (9.5)	38(9.3)
	OR (95% CI) ***	1	8.1 (3.3s19.6)	11.1 (4.5–27.6)	9.1 (4.5–18.6)	9.3 (4.9–17.7)
	ORa (95% CI) ***	1	12.0 (4.6–31.3)	15.2 (5.7–40.0)	14.1 (6.3–31.4)	13.9(6.7–29.0)
Manner of Washing ***	Shower/Bath n (%)	39 (76.5)	83 (98.8)	99 (99.0)	225 (100.0)	407(99.5)
	Towel/sponge n (%)	12 (23.5)	1 (1.2)	1 (1.0)	0 (0.0)	2(0.5)
	OR (95% CI) ***	1	25.5 (3.2–203.4)	30.4 (3.8–242.2)	ª	62.6(13.5–289.9)
	ORa (95% CI) ***	1	33.2 (4.0–273.2)	ª	ª	171.4(20.8–1411)
Time of Day	Bedtime n (%)	20 (39.2)	38 (45.2)	59 (57.8)	125 (56.1)	222(54.3)
	Other n (%)	31 (60.8)	46 (54.8)	43 (42.2)	98 (43.9)	187(45.7)
	OR (95% CI)	1	1.2 (0.6–2.5)	2.1 (1.0–4.2)	1.9 (1.0–3.6)	1.8(1.0–3.3)
	ORa (95% CI)	1	1.4 (0.6–2.9)	2.2 (1.1–4.6)	2.2 (1.1–4.2)	2.0(1.1–3.7)
Use Shower Gel ***	Yes n (%)	40 (78.4)	79 (92.9)	98 (96.1)	217 (96.4)	394(95.6)
	No/unknown n (%)	11 (21.6)	6 (7.1)	4 (3.9)	8 (3.6)	18(4.4)
	OR (95% CI) **	1	3.6 (1.2–10.5)	6.7 (2.9–22.4)	7.4 (2.8–19.7)	6.0(2.6–13.6)
	ORa (95% CI) ***	1	4.6 (1.5–14.1)	10.8 (2.8–42.1)	10.2 (3.6–28.2)	8.4(3.5–20.1)
Use sponge	No/unknown n (%)	15 (29.4)	14 (16.9)	25 (24.8)	84 (37.5)	123(30.1)
	Yes n (%)	36 (70.6)	69 (83.1)	76 (75.2)	140 (62.5)	285(69.9)
	OR (95% CI)	1	0.4 (0.2–1.1)	0.7 (0.3–1.6)	1.4 (0.7–2.7)	1.0(0.5–1.2)
	ORa (95% CI)	1	0.4 (0.1–1.0)	0.8 (0.3–1.7)	1.3 (0.7–2.6)	1.0(0.5–1.9)
Use bar of soap **	No/unknown n (%)	34 (66.7)	60 (74.1)	84 (84.0)	189 (85.5)	333(82.8)
	Yes n (%)	17 (33.3)	21 (25.9)	16 (16.0)	32 (14.5)	69(17.2)
	OR (95% CI) **	1	1.4 (0.6–3.0)	2.6 (1.1–5.7)	2.9 (1.4–5.9)	2.4(1.2–4.5)
	ORa (95% CI) **	1	1.3 (0.6–2.9)	2.7 (1.2–6.0)	2.9 (1.4–5.9)	2.2(1.1–4.3)
Use washbowl	No/unknown n (%)	38 (74.5)	76 (90.5)	97 (96.0)	219 (98.2)	392(96.1)
	Yes n (%)	13 (25.5)	8 (9.5)	4 (4.0)	4 (1.8)	16(3.9)
	OR (95% CI) ***	1	3.2 (1.2–8.5)	8.2 (2.5–27.0)	18.7 (5.7–60.4)	8.3(3.7–18.7)
	ORa (95% CI) ***	1	3.9 (1.4–10.7)	12.8 (3.3–48.5)	23.9 (7.1–79.9)	10.9(4.6–25.8)
Weekly frequency of hair Washing ***	>3 days n (%)	21 (41.2)	68 (80.0)	81 (80.2)	177 (80.1)	326(80.1)
	<2 days n (%)	30 (58.8)	17 (20.0)	20 (19.8)	44 (19.9)	81(19.1)
	OR (95% CI) ***	1	5.7 (2.6–12.3)	5.7 (2.7–12.1)	5.7 (3.0–10.9)	5.7(3.1–10.6)
	ORa (95% CI) ***	1	9.5 (4.0–22.4)	8.6 (3.8–19.6)	9.4 (4.4–19.6)	9.2(4.5–18.5)
Use Shampoo	Yes n (%)	48 (94.1)	80 (96.4)	100 (98.0)	217 (98.2)	397(97.8)
	No/unknown n (%)	3 (5.9)	3 (3.6)	2 (2.0)	4 (1.8)	9(2.2)
	OR (95% CI)	1	1.6 (0.3–8.5)	3.1 (0.5–19.3)	3.3 (0.7–15.6)	2.7(0.7–9.4)
	ORa (95% CI)	1	1.6 (0.3–8.9)	3.1 (0.4–19.4)	3.7 (0.7–17.7)	2.9(0.7–11.4)

CRC: Children in reception centers. CLF: children living on a family. OR: Odds Ratio. ORa: Odds Ratio adjusted for age and sex. ª OR calculation not applicable * *p* < 0.05, ** *p* < 0.01, *** *p* < 0.001.

**Table 4:** Comparison of hand hygiene variables between children in reception centers and those living with their family according to monthly income (N = 455).

Hand Washing		CRC n = 51	CLF Income <1000 € n = 83	CLF Income 1001 €–2000 € n = 97	CLF Income >2000 € n = 224	CLF Total n = 454
Daily frequency	>3 times n (%)	13 (25.5)	31 (36.5)	35 (35.0)	80 (35.7%)	146(35.7)
	≤3 times n (%)	38 (74.5)	54 (63.5)	65 (65.0)	144 (64.3)	263(64.3)
	OR (95% CI)	1	1.6 (0.7–3.6)	1.5 (0.7–3.3)	1.6 (0.8–3.2)	1.6(0.8–3.1)
	ORa (95% CI)	1	2.0 (0.9–4.4)	1.7 (0.8–3.8)	1.9 (0.9–3.9)	1.9(0.9–3.7)
Always before every meal *	Yes n (%)	5 (9.8)	47 (55.3)	53 (52.0)	116 (51.6)	216(52.4)
	No n (%)	46 (90.2)	38 (44.7)	49 (48.0)	109 (48.4)	196(47.6)
	OR (95% CI)*	1	11.3 (4.1–31.4)	9.9 (3.6–27.0)	9.7 (3.7–25.5)	10.1(3.9–26.0)
	ORa (95% CI) *	1	12.0 (4.3–33.3)	9.9 (3.6–27.2)	9.7 (3.7–25.5)	9.7(3.–25.2)
Always after defecating *	Yes n (%)	16 (31.4)	60 (70.6)	79 (77.5)	172 (77.1)	311(75.9)
	No n (%)	35 (68.6)	25 (29.4)	23 (22.5)	51 (22.9)	99(24.1)
	OR (95% CI) *	1	5.2 (2.4–11.1)	7.5 (3.5–15.9)	7.3 (3.7–14.4)	6.8(3.6–12.9)
	ORa (95% CI) *	1	9.5 (4.1–22.1)	11.2 (4.9–25.6)	12.8 (6.0–27.5)	11.5(5.6–23.7)
Always after urinating *	Yes n (%)	7 (13.7)	47 (55.3)	61 (59.8)	121 (53.8)	229(55.6)
	No n (%)	44 (86.3)	38 (44.7)	41 (40.2)	104 (46.2)	183(44.4)
	OR (95% CI) *	1	7.7 (3.1–19.2)	9.3 (3.8–22.7)	7.3 (3.1–16.9)	7.8(3.4–17.8)
	ORa (95% CI) *	1	10.6 (4.1–27.4)	11.9 (4.7–30.2)	10.1 (4.2–24.3)	11.1(4.7–26.4)
Always use soap *	Yes n (%)	20 (39.2)	63 (74.1)	91 (89.2)	181 (80.4)	335(81.3)
	No n (%)	31 (60.8)	22 (25.9)	11 (10.8)	44 (19.6)	77(18.7)
	OR (95% CI) *	1	4.4 (2.1–9.3)	12.8 (5.5–29.7)	6.3 (3.3–12.2)	6.7(3.6–12.4)
	ORa (95% CI) *	1	4.5 (2.1–9.6)	13.6 (5.7–32.3)	6.3 (3.3–12.2)	7.8(4.1–15.0)

CRC: children in reception centers. CLF: Children living on a family. OR: Odds Ratio. ORa: Odds Ratio adjusted for age and sex. * *p* < 0.001.

**Table 5:** Comparison of oral hygiene variables between children in reception centers and those living with their family according to monthly income (N = 456).

Oral Hygiene		CRC n = 51	CLF Income <1000 € n = 83	CLF Income 1001 €–2000 € n = 98	CLF Income >2000 € n = 224	CLF Total n = 454
Daily tooth brushing frequency **	≥twice n (%)	17 (33.3)	57 (67.1)	75 (73.5)	184 (82.1)	316(76.9)
	<twice n (%)	34 (66.7)	28 (32.9)	27 (26.5)	40 (17.9)	95(23.1)
	OR (95 CI) **	1	4.0 (1.9–8.5)	5.5 (2.6–11.5)	9.2 (4.6–18.0)	6.6(3.5–12.4)
	ORa (95% CI) **	1	4.9 (2.2–10.6)	6.4 (2.9–13.7)	11.8 (5.7–24.3)	8.6(4.2–16.3)
Dentist visits during the previous year **	Yes n (%)	17 (34.0)	68 (82.9)	90 (90.9)	203 (91.4)	361(89.6)
	No n (%)	33 (66.0)	14 (17.1)	9 (9.1)	19 (8.6)	42(10.4)
	OR (95% CI) **	1	9.4 (4.1–21.4)	19.4 (7.8–47.7)	20.7 (9.7–43.9)	16.6(8.5–32.4)
	ORa (95% CI) **	1	10.0 (4.3–23.0)	18.5 (7.5–45.7)	20.7 (9.7–43.9)	19.0(9.4–38.4)
Brush when getting up in the morning **	Yes n (%)	9 (17.6)	53 (62.4)	55 (54.5)	108 (48.0)	216(52.6)
	No n (%)	42 (82.4)	32 (37.6)	46 (45.5)	117 (52.0)	195(47.4)
	OR (95% CI) **	1	7.7 (3.3–17.9)	5.5 (2.4–12.6)	4.3 (2.0–9.2)	5.1(2.4–10.8)
	ORa (95% CI) **	1	7.8 (3.3–18.2)	5.8 (2.5–13.3)	4.3 (2.0–9.2)	5.6(2.6–12.1)
Brush after every main meal **	Yes n (%)	6 (11.8)	37 (44.6)	51 (51.0)	102 (45.3)	190(46.6)
	No n (%)	45 (88.2)	46 (55.4)	49 (49.0)	123 (54.7)	218(53.4)
	OR (95% CI) **	1	6.0 (2.3–15.6)	7.8 (3.0–19.9)	6.2 (2.5–15.1)	6.5(2.7–15.6)
	ORa (95% CI) **	1	6.3 (2.4–16.4)	7.6 (2.9–19.6)	6.2 (2.5–15.1)	6.7(2.7–16.2)
Wash before going to bed at night **	Yes n (%)	13 (25.5)	64 (77.1)	86 (85.1)	191 (85.7)	341(83.8)
	No n (%)	38 (74.5)	19 (22.9)	15 (14.9)	32 (14.3)	66(16.2)
	OR (95% CI) **	1	9.8 (4.3–22.1)	16.7 (7.2–38.6)	17.4 (8.3–36.2)	15.1(7.6–29.8)
	ORa (95% CI) **	1	12.2 (5.1–28.8)	23.8 (9.6–58.4)	23.0 (10.4–50.9)	20.(9.5–42.2)
Use toothbrush **	Yes n (%)	41 (80.4)	84 (98.8)	102 (100.0)	223 (99.6)	409(99.5)
	No/unknown n (%)	10 (19.6)	1 (1.2)	0 (0.0)	1 (0.4)	2(0.5)
	OR (95% CI) **	1	20.4 (2.5–165.5)	ª	54.3 (6.7–436.4)	49.8(10.5235.4)
	ORa (95% CI) **	1	20.0 (2.4–161.6)	ª	54.3 (6.7–436.4)	55.7(11.3–272.8)
Use toothpaste **	Yes n (%)	40 (78.4)	84 (98.8)	100 (98.0)	222 (99.1)	406(98.8)
	No/unknown n (%)	11 (21.6)	1 (1.2)	2 (2.0)	2 (0.9)	5(1.2)
	OR (95% CI) **	1	23.1 (2.8–185.1)	13.7 (2.9–64.8)	30.5 (6.5–142.9)	22.3(7.3–67.4)
	ORa (95% CI) **	1	22.5 (2.8–180.8)	13.2 (2.7–62.2)	30.5 (6.5–142.9)	23.9(7.7–74.6)
Use mouthwash	Yes n (%)	9 (17.6)	31 (36.5)	45 (44.6)	81 (36.5)	157(38.5)
	No/unknown n (%)	42 (82.4)	54 (63.5)	56 (55.4)	141 (63.5)	251(61.5)
	OR (95% CI) *	1	2.6 (1.1–6.2)	3.7 (1.6–8.5)	2.6 (1.2–5.7)	2.9(1.3–6.1)
	ORa (95% CI) **	1	3.7 (1.5–9.0)	5.0 (2.1–11.9)	3.6 (1.6–8.1)	3.9(1.8–8.7)
Use dental floss *	Yes n (%)	3 (5.9)	17 (20.0)	30 (29.4)	45 (20.4)	92(22.5)
	No/unknown n (%)	48 (94.1)	68 (80.0)	72 (70.6)	176 (79.6)	316(77.5)
	OR (95% CI) *	1	4.0 (1.1–14.4)	6.6 (1.9–23.0)	4.0 (1.2–13.7)	4.6(1.4–15.3)
	ORa (95% CI) *	1	4.1 (1.1–14.8)	6.7 (1.9–23.3)	4.0 (1.2–13.7)	4.7(1.4–15.7)
Use toothpick **	No/unknown n (%)	32 (62.7)	72 (84.7)	79 (77.5)	208 (93.7)	359(87.8)
	Yes n (%)	19 (37.3)	13 (15.3)	23 (22.5)	14 (6.3)	50(12.2)
	OR (95% CI) **	1	3.2 (1.4–7.4)	2.0 (0.9–4.2)	8.8 (4.0–19.3)	4.2(2.2–8.0)
	ORa (95% CI) **	1	3.2 (1.4–7.4)	2.2 (1.0–4.6)	9.4 (4.2–20.8)	4.2(2.1–8.1)
Type of toothbrush **	Electrical n (%)	3 (5.9)	33 (38.8)	42 (41.2)	93 (41.7)	168(41.0)
	Manual n (%)	48 (94.1)	52 (61.2)	60 (58.8)	130 (58.3)	242(59.0)
	OR (95% CI) **	1	10.1 (2.9–35.2)	11.2 (3.2–38.3)	11.4 (3.4–37.8)	11.1(3.4–36.2)
	ORa (95% CI) **	1	10.5 (3.0–36.7)	11.0 (3.2–37.9)	11.4 (3.4–37.8)	11.3(3.4–37.4)
Share toothbrush with others **	Yes n (%)	9 (17.6)	0 (0.0)	2 (2.0)	2 (0.9)	4(1.0)
	No n (%)	42 (82.4)	81 (100.0)	97 (98.0)	221 (99.1)	399(99.0)
	OR (95% CI) **	1	ª	10.3 (2.1–50.1)	23.6 (4.9–113.5)	21.3(6.3–72.3)
	ORa (95% CI) **	1	ª	9.9 (2.0–48.1)	23.6 (4.9–113.5)	25.9(7.4–90.6)
Tooth brushing duration **	1–3 min n (%)	17 (33.3)	60 (70.6)	68 (66.7)	152 (67.6)	280(64.1)
	< 1 min n (%)	34 (66.7)	25 (29.4)	34 (33.3)	73 (32.4)	132(32.0)
	OR (95% CI) **	1	4.8 (2.2–10.1)	4.0 (1.9–8.1)	4.1 (2.1–7.9)	4.2(2.2–7.8)
	ORa (95% CI) **	1	6.6 (3.0–14.6)	5.0 (2.3–10.6)	5.3 (2.7–10.5)	5.5(2.8–10.7)

CRC: children in reception centers. CLF: children living in a family. OR: Odds Ratio. ORa: Odds Ratio adjusted for age and sex. ª OR calculation not applicable * *p* < 0.01, ** *p* < 0.001.

**Table 6:** Comparison of socio–educational variables between children in reception centers and those living with their family according to monthly income (N = 455).

Socio–Educational Variables			CRC n = 51	CFL Income <1000 € n = 82	CFL Income 1001 €–2000 € n = 97	CFL Income >2000 € n = 225	CLF Total n = 454
Influences on body hygiene	Father, mother, or other relatives **	Yes n (%) No n (%)	37(72.5) ^‡^ 14(27.5) ^‡^	79(92.9) 6(7.1)	94(93.1) 7(6.9)	204(90.7) 21(9.3)	377(91.7) 34(8.3)
	Friends	Yes n (%) No n (%)	2(3.9) 49(96.1)	2(2.4) 81(97.6)	1(1.0) 98(99.0)	2(0.9) 222(99.1)	5(1.2) 401(98.8)
	Neighbors	Yes n (%) No n (%)	2(3.9) 49(96.1)	3(3.6) 81(96.4)	0(0.0) 99(100.0)	2(0.9) 222(99.1)	5(1.2) 402(98.8)
	Radio, television, Internet *	Yes n (%) No n (%)	7(13.7) ^‡^ 44(86.3) ^‡^	7(8.3) 77(91.7)	3(3.1) 95(96.9)	10(4.5) 214(95.5)	20(4.9) 386(95.1)
	Teachers	Yes n (%) No n (%)	13(25.5) 38(74.5)	9(10.7) 75(89.3)	12(12.2) 86(87.8)	33(14.7) 191(85.3)	54(13.3) 352(86.7)
	Nurses Physicians	Yes n (%) No n (%)	13(25.5) 38(74.5)	32(38.1) 52(61.9)	33(33.0) 67(67.0)	84(37.5) 140(62.5)	149(36.5) 259(63.5)
	Self–learning	Yes n (%) No n (%)	11(21.6) 40(78.4)	9(11.1) 72(88.9)	11(11.1) 88(88.9)	22(9.8) 202(90.2)	42(10.4) 362(89.6)
	Unknown	Yes n (%) No n (%)	1(2.0) 50(98.0)	5(6.8) 68(93.2)	2(2.2) 87(97.8)	6(2.7) 216(97.3)	13(3.4) 371(96.6)
Social rejection	Rejected for being dirty **	Never n (%) Occasionally n (%) Several times n (%)	30(58.8) ^‡^ 4(7.8) 17(33.3) ^‡^	76(90.5) 7(8.3) 1(1.2)	98(96.1) ^‡^ 4(3.9) 0(0.0) ^‡^	214(95.5) ^‡^ 10(4.5) 0(0.0) ^‡^	388(94.6) 21(5.1) 1(0.2)
	Rejected for smelling bad **	Never n (%) Occasionally n (%) Several times n (%)	26(51.0) ^‡^ 7(13.7) 18(35.3) ^‡^	75(89.3) 8(9.5) 1(1.2)	93(91.2) 8(7.8) 1(1.0) ^‡^	204(91.1) ^‡^ 19(8.5) 1(0.4) ^‡^	372(90.7) 35(8.5) 3(0.7)
Reasons for hygiene	To be healthy	Yes n (%) No n (%)	46(90.2) 5(9.8)	77(90.6) 8(9.4)	87(87.0) 13(13.0)	200(89.3) 24(10.7)	364(89.0) 45(11.0)
	To not smell bad	Yes n (%) No n (%)	49(96.1) 2(3.9)	78(91.8) 7(8.2)	89(87.3) 13(12.7)	208(93.3) 15(6.7)	375(91.5) 35(8.5)
	To not be rejected by friends **	Yes n (%) No n (%)	46(90.2) ^‡^ 5(9.8) ^‡^	49(58.3) ^‡^ 35(41.7) ^‡^	36(36.0) ^‡^ 64(64.0) ^‡^	81(36.3) ^‡^ 142(63.7) ^‡^	166(40.8) 241(59.2)
	To not be punished at home	Yes n (%) No n (%)	17(33.3) 34(66.7)	24(28.2) 61(71.8)	24(24.0) 76(76.0)	59(26.5) 164(73.5)	107(26.2) 301(73.0)
	To feel good	Yes n (%) No n (%)	49(96.1) 2(3.9)	81(95.3) 4(4.7)	96(95.0) 5(5.0)	211(94.2) 13(5.8)	388(94.6) 22(5.4)

CRC: children in reception centers. CFL: children living in a family * *p* < 0.05. ** *p* < 0.001. ^^‡^^ Significant cell.
